# Using General-purpose Sentiment Lexicons for Suicide Risk Assessment in Electronic Health Records: Corpus-Based Analysis

**DOI:** 10.2196/22397

**Published:** 2021-04-13

**Authors:** André Bittar, Sumithra Velupillai, Angus Roberts, Rina Dutta

**Affiliations:** 1 Institute of Psychiatry, Psychology and Neuroscience King’s College London London United Kingdom; 2 South London and Maudsley NHS Foundation Trust London United Kingdom

**Keywords:** psychiatry, suicide, suicide, attempted, risk assessment, electronic health records, sentiment analysis, natural language processing, corpus linguistics

## Abstract

**Background:**

Suicide is a serious public health issue, accounting for 1.4% of all deaths worldwide. Current risk assessment tools are reported as performing little better than chance in predicting suicide. New methods for studying dynamic features in electronic health records (EHRs) are being increasingly explored. One avenue of research involves using sentiment analysis to examine clinicians’ subjective judgments when reporting on patients. Several recent studies have used general-purpose sentiment analysis tools to automatically identify negative and positive words within EHRs to test correlations between sentiment extracted from the texts and specific medical outcomes (eg, risk of suicide or in-hospital mortality). However, little attention has been paid to analyzing the specific words identified by general-purpose sentiment lexicons when applied to EHR corpora.

**Objective:**

This study aims to quantitatively and qualitatively evaluate the coverage of six general-purpose sentiment lexicons against a corpus of EHR texts to ascertain the extent to which such lexical resources are fit for use in suicide risk assessment.

**Methods:**

The data for this study were a corpus of 198,451 EHR texts made up of two subcorpora drawn from a 1:4 case-control study comparing clinical notes written over the period leading up to a suicide attempt (cases, n=2913) with those not preceding such an attempt (controls, n=14,727). We calculated word frequency distributions within each subcorpus to identify representative keywords for both the case and control subcorpora. We quantified the relative coverage of the 6 lexicons with respect to this list of representative keywords in terms of weighted precision, recall, and F score.

**Results:**

The six lexicons achieved reasonable precision (0.53-0.68) but very low recall (0.04-0.36). Many of the most representative keywords in the suicide-related (case) subcorpus were not identified by any of the lexicons. The sentiment-bearing status of these keywords for this use case is thus doubtful.

**Conclusions:**

Our findings indicate that these 6 sentiment lexicons are not optimal for use in suicide risk assessment. We propose a set of guidelines for the creation of more suitable lexical resources for distinguishing suicide-related from non–suicide-related EHR texts.

## Introduction

### Background

The World Health Organization reports that suicide accounts for 1.4% of all deaths globally and is the 18th leading cause of death worldwide [1]. Prior history of suicide attempts is the most robust risk factor for completed suicide, and those requiring hospitalization are at the most serious end of the spectrum [2]. However, current methods for assessing a patient’s risk of attempting suicide are reported to perform little better than chance [3]. Therefore, new methods to understand dynamic features from electronic health records (EHRs) before a hospitalized suicide attempt, distinguishing such periods from clinical narratives at other times, would be of potential clinical utility [4].

EHRs contain structured patient data (eg, age, sex, and ethnicity) and unstructured text that make up the clinical narrative (eg, out-patient letters, event notes from meetings and phone calls with patients or carers, and discharge summaries). Unstructured text is of particular importance in mental health, as much of what is recorded about patients follows face-to-face assessments by clinicians, whose observations and judgments about a patient’s experiences and presentation are inevitably influenced by their own training, experience, and implicit biases, and these judgments have a degree of subjectivity when they record this in the clinical narrative [5].

The automatic identification and analysis of subjective judgments in text is known as sentiment analysis [6,7]. This process typically involves the classification of words as expressing either positive or negative polarity, and numerous resources have been developed for this task in nonclinical domains, such as customer reviews [8-11] and social media [12-14]. Research efforts have also focused on the analysis of sentiment within health care–related texts, such as patient feedback forms [15,16], online forums [17], and social networks [18,19].

Recent work has sought to assess the utility of sentiment lexicons for the analysis of subjective judgments in clinical narratives. McCoy et al [20] used a general-domain sentiment analysis tool to extract word polarity features to model the risk of readmission and mortality. The same tool was later used to examine the correlation between word polarity and the risk of suicide attempts [21]. Most recently, Weissman et al [22] carried out a thorough evaluation of six general-domain sentiment analysis tools in predicting the risk of in-hospital mortality of patients in intensive care, tracking the progression of sentiment in clinical notes over time. They concluded that general-domain sentiment tools are not suited to the processing of clinical texts and that domain-specific resources need to be developed. Work in this direction is beginning to emerge [23-25].

These studies have mostly focused on testing the correlation between automatically extracted sentiment values and specific clinical outcomes. However, to our knowledge, there has been no close examination of the terms mapped by general-domain sentiment analysis tools when applied to clinical texts.

### Objectives

Focusing on words with negative and positive polarity, we aimed to determine the coverage of 6 general-purpose sentiment lexicons when applied to a corpus of EHR texts of 2 groups of patients seen by mental health services: (1) patients who had attempted suicide and were hospitalized (cases) and (2) patients with no history of attempted suicide (controls). Adopting methods used in corpus linguistics, we first sought to identify the words that are most representative of the clinical narratives of cases and controls. We then aimed to test the coverage of each sentiment lexicon by comparing these 2 sets of representative words. We sought to ascertain the extent to which these 2 sets of representative words contained general-purpose sentiment words and to what extent these 2 sets contained additional sentiment words not included in the general-purpose lexicons.

## Methods

### Corpus Analysis

#### Clinical Cohort

We studied deidentified EHRs of over 250,000 patients from the South London and Maudsley National Health Service Foundation Trust using the Clinical Record Interactive Search (CRIS) database, comprising over 3.5 million text documents [26]. CRIS has been linked with national hospital admission data within a secure *safe haven*, allowing hospital admission information to be extracted. The deidentified CRIS database has received ethical approval for secondary analysis: Oxford REC C, reference 18/SC/0372. Access is granted upon request to authorized researchers working on projects that have received prior approval from the CRIS Oversight Committee. The data presented in this study can be viewed within the secure system firewall.

Our data set was derived from the EHRs of 17,640 patients. It consisted of 4235 suicide attempt–related (case) admissions and 16,940 nonsuicide attempt–related (control) admissions, sampled according to a 1:4 case-control ratio. Cases were defined as any admission (acute physical or specialist mental health) where there was a suicide attempt (indicated by any of the following codes from the International Classification of Diseases (ICD-10): X6*, X7*, X80-4*, Y1*, Y2*, Y30-4*, and Y87*) with the admission lasting at least 24 hours. Admissions starting on or after April 1, 2006, and ending before or including March 31, 2017, were considered. Case admissions that had at least one document in the 30 days up to and including the date of the suicide attempt were retained. We also removed admissions with empty documents (text from scanned documents is not always available in CRIS), resulting in a total of 4235 suicide-related admissions. Controls did not have any of the specified ICD-10 codes in the given period, were matched by sex, had to be alive at the admission start date of the corresponding case, and were matched to the same age group (5-year age bands: <16, 16-19, 20-24 to 80-84, and >85 years). Each control also had at least one document in the 30 days up to and including the date of the suicide attempt of the matched case. The controls were chosen to be representative (in terms of age and sex) of the population from which the cases were drawn, and the ratio was based on the epidemiological principle that little statistical power is gained by further increasing the number of controls beyond approximately 4 per case [27]. The key descriptive characteristics of the cohort are presented in Table 1.

**Table 1:** Cohort patient- and admission-level statistics.

Unit of observation		Cases	Controls
**Patients,** **n (%)**		2913 (16.51)	14,727 (83.49)
	Female	1730 (59.39)	8971 (60.92)
	Male	1183 (40.61)	5756 (39.08)
**Admissions,** **n (%)**		4235 (20.00)	16,940 (80.00)
	Female	2598 (61.35)	10,392 (61.35)
	Male	1637 (38.65)	6548 (38.65)
Age (years), mean (SD)		34.4 (15.3)	34.4 (15.4)

#### EHR Corpus

Our corpus comprised all EHR texts for each of the 2 subgroups in our clinical cohort: (1) suicidal case admissions and (2) nonsuicidal controls.

Our use of a 1:4 case-control study design for admissions means we expect a disparity in document number and word count between subcorpora. However, there are only 77.92% (55,643/71,404) more control documents (n=127,047) than case documents (n=71,404), rather than the 300% difference that might be expected for 1:4 sampling of random patients. Following data preprocessing (refer to the *Data Preparation* subsection), the mean lexical word count for case documents (n=117.4) is higher than that for control documents (n=103.9), so that the overall word (token) count ratio is not 1:4 but approximately 1:1.6, whereas the mean unique word (type) count ratio is approximately 1.5. The basic descriptive statistics for the corpus are shown in Table 2. The distribution of documents per patient followed a non-normal distribution, as shown in Multimedia Appendix 1.

**Table 2:** Electronic health record corpus descriptive statistics.

Unit of observation	Cases	Controls	Total
Word tokens, n	8,385,643	13,198,250	21,583,893
Word types, n	109,024	162,696	206,866
Type-token ratio^a^, %	1.30	1.23	0.96
Documents, n	71,404	127,047	198,451
Number of words per document, mean (SD)	117.4 (219.1)	103.9 (252.7)	108.8 (241.3)

^a^Type-token ratio = number of word types / number of word tokens × 100.

#### Data Preparation

All texts were preprocessed using the Natural Language Processing (NLP) library spaCy (v2.0.12) [28], applying the following steps: word tokenization, part-of-speech tagging, and lemmatization (to use the base form of words). We removed stop words using the Natural Language ToolKit [29] stop words list for English and lowercased all words for our analyses. All codes were made available on GitHub [30].

#### Identifying Representative Keywords

To answer our questions concerning the coverage of each lexicon, we adopted methods based on word frequency distributions, commonly used in corpus linguistics, as described further in Multimedia Appendix 1 (C) [31-34]. We first determined which *keywords* were most *representative* of each subcorpus (suicidal case admission texts and nonsuicidal control texts) by calculating the relative word frequency ratios between subcorpora. Following recommendations from previous research in corpus linguistics [31-33] and given the non-normal distribution of documents between patients, we then applied the nonparametric Mann-Whitney *U* test to determine the statistical significance of word frequency differences (*FreqDiff (w)* for a given word w) between subcorpora. We only retained words that occurred in both the case and control subcorpora, leaving a total of 64,854 unique token types. Words appearing in only one or other subcorpora were relatively infrequent compared with those that were common to both subcorpora. For example, the most frequent case-only keywords were identifying initials, with a maximum frequency of 20.2 words per million (wpm), whereas the most frequent control-only keywords were persons’ names, with a maximum frequency of 34.4 wpm.

### Sentiment Lexicon Analysis

#### Sentiment Lexicons

We examined six different sentiment lexicons that were developed for nonclinical domains. Various dimensions of sentiment and affect have been studied, including emotion, valence-arousal-dominance, and polarity. We focused solely on lexicons that represent this last aspect, that is, negative and positive sentiment polarity. Along with assigning negative and positive polarity, some sentiment analysis tools also assign a value for words that do not convey semantic polarity (ie, *neutral* words). However, we only considered words that express positive and negative sentiments, as not all the lexicons in this study contain neutral terms. Therefore, we filtered out any neutral words. Furthermore, for the sake of comparison, we only examined binary sentiment values rather than degree scores, which only some lexicons provide. We selected the following lexicons for this study: AFINN [35], the NRC Emotion Lexicon (commonly known as EmoLex) [36], Linguistic Inquiry and Word Count (LIWC) [37], the Opinion lexicon [9], the Pattern lexicon [38], and SentiWordNet [39]. The lexicons differ in terms of the forms they contain (words, lemmas, and regular expressions). We applied each one *as-is* to the appropriately preprocessed corpus (eg, words or lemmas) to compare them, as they have been used in other studies. We provide details of the lexicons, including preprocessing and filtering, in Multimedia Appendix 1 (B) [9,35-44]. Table 3 summarizes some of the main characteristics of each of these lexicons, including size before (original size) and after (filtered size) filtering out neutral entries.

**Table 3:** Characteristics of the 6 sentiment lexicons.

Lexicon	Source	Automatic term selection	Intended domain	Term type	Original size (entries), n	Filtered size (number of entries), n (%)
AFINN	Various web-based word lists	No	Microblogs	Word forms	3478	3478 (100.00)
EmoLex	Macquarie Thesaurus, General Inquirer, WordNet	No	General	Word forms	14,182	5555 (39.17)
LIWC^a^	Various dictionaries and thesauruses	No	Personal narratives	Word forms and regular expressions	1371	1371 (100.00)
Opinion	Web crawl of product reviews	Yes	Product reviews	Word forms	6789	6789 (100.00)
Pattern	Subset of WordNet	No	Product reviews	Lemmas+POS^b^	2896	2293 (79.18)
SentiWordNet	WordNet	Yes	General	Synset Lemmas+POS	117,659	39,746 (33.78)

^a^LIWC: Linguistic Inquiry and Word Count.

^b^POS: part of speech.

#### Lexicon Coverage

We assessed the coverage of each lexicon in three different ways:

1. *Global coverage*: The percentage of sentiment-bearing lexical entries that appeared in the list of (unique) words for each subcorpus. Further details are provided in Multimedia Appendix 1 (D).
2. *Keyword coverage*: The proportion of case and control keywords covered by the sentiment-bearing terms of a lexicon. First, we calculated the percentage of keywords identified by each lexicon for each subcorpus. Second, we used metrics common to information retrieval, namely, weighted precision (P_w_), recall (R_w_), and *F* score (F_w_), which we calculated for each lexicon across the unordered set of all keywords, using word ranking as the weighting. Details of our calculations, including formulae, are provided in Multimedia Appendix 1 (D). A lexicon’s precision shows how many case keywords it correctly identifies as a proportion of all the keywords it contains. The inclusion of control keywords in a lexicon, therefore, penalizes precision. In contrast, recall indicates the number of case keywords that the lexicon correctly identifies from the entire list of case keywords. The absence of case keywords from a lexicon results in a penalty on recall. *F*score provides a combination of the preceding 2 metrics and an overall quantified evaluation of a lexicon’s keyword coverage.
3. *Sentiment coverage*: The sentiment polarity (positive or negative) that lexicons assigned to matched keywords for each subcorpus.

## Results

### Corpus Analysis

The step of generating representative keywords for each subcorpus (refer to the *Corpus analysis* subsection) resulted in a list of 3382 keywords. Sorted by decreasing the frequency difference, the top words (with *FreqDiff*>0) are representative of the suicidal case subcorpus (2360 keywords). Similarly, sorting in ascending order, top words (with *FreqDiff*<0) are representative of the nonsuicidal control subcorpus (1022 keywords). Table 4 shows the 10 top-ranking keywords for each subcorpus. In this table, we show each word’s rank as well as its frequency in the whole corpus, the frequency difference between case and control subcorpora, and the frequency ratio for the word across the subcorpora. We provide a similar list of the top 100 keywords in Multimedia Appendix 2.

**Table 4:** Ranked keyword list for suicidal case and nonsuicidal control subcorpora.

Suicidal case keywords					Nonsuicidal control keywords				
Rank	Word	Freq^a^ (words per million)	Freq diff^b^	Freq ratio^c^	Rank	Word	Freq (words per million)	Freq diff	Freq ratio
1	QQQQQ^d^	9779.1	3545.7	1.6	1	ZZZZZ^d^	35657.1	−3801.4	1.1
2	self	4278.5	2060.9	1.9	2	mental	3092.5	−1242.5	1.4
3	harm	2916.2	1673.4	2.4	3	mr	1197.9	−1138.1	2.0
4	ward	5554.7	1597.1	1.4	4	appointment	1583.5	−1124.5	1.7
5	overdose	1717.0	1392.8	5.3	5	medication	3756.5	−1017.4	1.3
6	staff	5670.0	1389.4	1.3	6	health	2282.2	−771.1	1.3
7	suicidal	2072.5	1256.2	2.5	7	please	1305.9	−703.6	1.5
8	said	5725.4	1137.7	1.3	8	state	1640.3	−694.4	1.4
9	alcohol	2276.2	1102.4	1.9	9	service	1190.6	−678.1	1.6
10	a&e	1534.1	1089.5	3.5	10	road	729.3	−596.2	1.8

^a^Freq: word frequency.

^b^Freq diff: frequency difference.

^c^Freq ratio: frequency ratio between subcorpora.

^d^Masking strings created by the electronic health record deidentification process: QQQQQ for relative or close contact identifiers and ZZZZZ for patient identifiers.

For the suicidal case subcorpus, the top keyword “QQQQQ” is a placeholder for anonymized names of relatives or close contacts of the patient created by a bespoke deidentification algorithm used in CRIS [45]. This could indicate concerns of relatives or carers being reported to staff over the patient’s status. Other top keywords directly relate to the theme of suicide attempts (*overdose*, *suicidal*, and *a&e* [accident and emergency]). The frequency ratio indicates that *overdose* is over 5 times and *a&e* is over 3.5 times more frequent in the case subcorpus than in the control subcorpus. Other words relate to hospitalization (*ward* and *staff*) and self-harm (*self* and *harm*).

Visual inspection shows that *self* and *harm* frequently co-occur in noun phrases such as *harm to self* and *self-harm* (which was incorrectly segmented into 2 tokens by the tokenizer). Furthermore, *harm* also occurs with reflexive pronouns, for example, *harm himself/herself*, also referencing self-harm events. *Alcohol* is also clinically relevant because both chronic alcohol use disorders and acute use of alcohol confer risk for attempted suicide.

In contrast, for the control subcorpus, the top keyword “ZZZZZ” is a placeholder for anonymized patient identifiers. These top keywords are more generic terms that may be found in most types of clinical notes (eg, *mental*, *health*, and *state*) and some are likely to be derived from correspondence (eg, *mr*, *appointment*, and *please*). Although the top control keywords are significantly more frequent than those in the case subcorpus, the frequency difference and ratio are globally less marked than for case keywords. The median absolute frequency difference (*FreqDiff*) for the top 10 control keywords is 894.2, compared with 1391.1 for cases. The corresponding median frequency ratios (*FreqRatio*) are 1.90 for cases and 1.45 for controls. This indicates that keywords for suicide-related texts are more strongly representative of the case subcorpus than the keywords for the control subcorpus. This may reflect the fact that cases have a distinct unifying feature of being included for their hospitalized suicide attempt, whereas control admissions were from any period as long as they did not precede a suicide attempt. It should be noted that no suppositions about the sentiment associated with these keywords were made.

### Sentiment Lexicon Analysis

We first assessed the global coverage of sentiment lexicons (refer to Multimedia Appendix 1 (E) for details). The figures for global coverage are summarized in Table 5.

**Table 5:** Term type and token counts for each lexicon in case and control subcorpora and whole corpus. Percentages for control words are shown as (raw/adjusted). Figures are in descending order of lexicon (filtered) size.

Lexicon	Filtered size	Word types			Word tokens		
		Case, n (%)	Control, n (%)	Whole corpus, n (%)	Case, n (%)	Control, n (%)	Whole corpus, n (%)
SentiWordNet	39,746	9843 (9.02)	12,429 (7.64/5.12)	13,373 (6.46)	4,234,058 (50.49)	8,603,932 (65.19/41.42)	12,837,990 (59.48)
Opinion	6789	3111 (2.85)	3662 (2.25/1.51)	3821 (1.85)	979,804 (11.68)	1,959,007 (14.84/9.43)	2,938,811 (13.62)
EmoLex	5555	3733 (3.42)	4260 (2.62/1.75)	4426 (2.14)	1,456,097 (17.36)	2,869,472 (21.74/13.81)	4,325,569 (20.04)
AFINN	3478	2529 (2.32)	2781 (1.71/1.15)	2845 (1.37)	1,274,283 (15.20)	2,532,261 (19.19/12.19)	3,806,544 (17.64)
Pattern	2293	1101 (1.01)	1243 (0.76/0.51)	1296 (0.63)	910,369 (10.86)	1,957,386 (14.83/9.42)	2,867,755 (13.29)
LIWC^a^	1371	3708 (3.40)	5824 (3.58/2.40)	6269 (3.03)	620,546 (7.40)	1,830,216 (13.87/8.81)	2,450,762 (11.35)

^a^LIWC: Linguistic Inquiry and Word Count.

SentiWordNet, by far the largest lexicon, has the widest coverage of approximately 60% of all tokens (6.46% types) in the entire corpus. The pattern has the lowest word-type coverage for both subcorpora and the whole corpus (0.63%). Although LIWC has the fewest lexical entries (1371), its use of regular expressions that capture multiple word forms means it maps more individual word types (but has the lowest coverage of tokens, 11.35% on the whole corpus). Despite having approximately 1200 and 3300 fewer entries than Opinion, respectively, EmoLex and AFINN both have a substantially higher coverage of word tokens over the larger lexicon. EmoLex also has a slightly higher coverage of token types. This may be a consequence of the manner in which these lexicons were constructed and the sources from which they were derived. We review this issue in the *Discussion* section.

With the exception of LIWC, all lexicons show higher coverage of word types in the case subcorpus than in the control subcorpus. The same trend was observed when considering the adjusted percentages for word tokens. This suggests that there is generally more *sentiment* (as defined in these lexicons) expressed in the case subcorpus than in the control subcorpus, assuming an artificial scenario in which there are an equal number of words of each. However, if no adjustment for word frequency disparities across subcorpora is made, the opposite tendency is observed for all lexicons.

This notion of coverage does not take into account the representativeness of the words in question. To capture this crucial characteristic, we examined the proportion of keywords (word types) from each subcorpus containing each lexicon (keyword coverage; refer to the *Corpus Analysis* subsection and Multimedia Appendix 1 [D]). The overall proportional coverage of keywords is shown in Table 6.

**Table 6:** Case and control keywords that appear in each sentiment lexicon, in descending order of lexicon (filtered) size. The total number of keywords for the case subcorpus is 2360 and for the control subcorpus is 1022.

Lexicon	Filtered size	Case, n (%)	Control, n (%)
SentiWordNet	39,746	604 (25.6)	231 (22.6)
Opinion	6789	192 (8.1)	60 (5)
EmoLex	5555	277 (11.7)	117 (11.4)
AFINN	3478	238 (10.1)	74 (7)
Pattern	2293	115 (4.9)	39 (3)
LIWC^a^	1371	181 (7.7)	48 (4)

^a^LIWC: Linguistic Inquiry and Word Count.

As with global coverage, keyword coverage is correlated with lexicon size, with LIWC being the exception. Again, when examining only the most representative words for each subcorpus, Opinion, the second largest resource, has substantially lower coverage than both EmoLex and AFINN, which are smaller in size, the latter resource numbering only half as many keywords among its entries.

Evaluating the lexicons from an information retrieval perspective revealed the extent to which each lexicon strikes a balance between the inclusion of case keywords and the exclusion of control keywords, accounting for the representativeness of the words identified. As shown in Table 7, all lexicons provided reasonable weighted precision (0.53-0.72). However, weighted recall and weighted F-score, which varied substantially across lexicons, were very low (0.04-0.36).

**Table 7:** Weighted metrics for each lexicon in descending order of weighted F score.

Lexicon	Weighted precision	Weighted recall	Weighted *F* score
SentiWordNet	0.68	0.36	0.47
EmoLex	0.68	0.18	0.29
AFINN	0.72	0.15	0.25
Opinion	0.68	0.11	0.18
LIWC^a^	0.69	0.10	0.17
Pattern	0.53	0.04	0.07

^a^LIWC: Linguistic Inquiry and Word Count.

These results show that, of all the lexicons we tested, SentiWordNet provides the best balance between precision and recall over keywords from the 2 subcorpora. Owing to its size, it obtained the highest recall. This indicates that it contains more of the most highly ranked case keywords than the other lexical resources. It also achieved precision on par with the other lexicons, indicating that the words it identifies are often high-ranking keywords from the suicide-related case subcorpus. The pattern lexicon achieved significantly lower results in terms of weighted precision and recall than all other lexicons, despite being larger than some of these. This suggests that its included sentiment terms are of a somewhat different nature and do not contribute a clear signal for distinguishing representative case keywords from control keywords.

Overall, as tools for distinguishing suicide-related from nonsuicide-related clinical notes, this evaluation, in particular the recall figures, shows that the most representative keywords in both subcorpora are not sentiment bearing, as defined in all these lexicons, thus indicating that there is a need for further analysis of the representative subcorpus keywords to better understand their characteristics.

**Figure 1:**
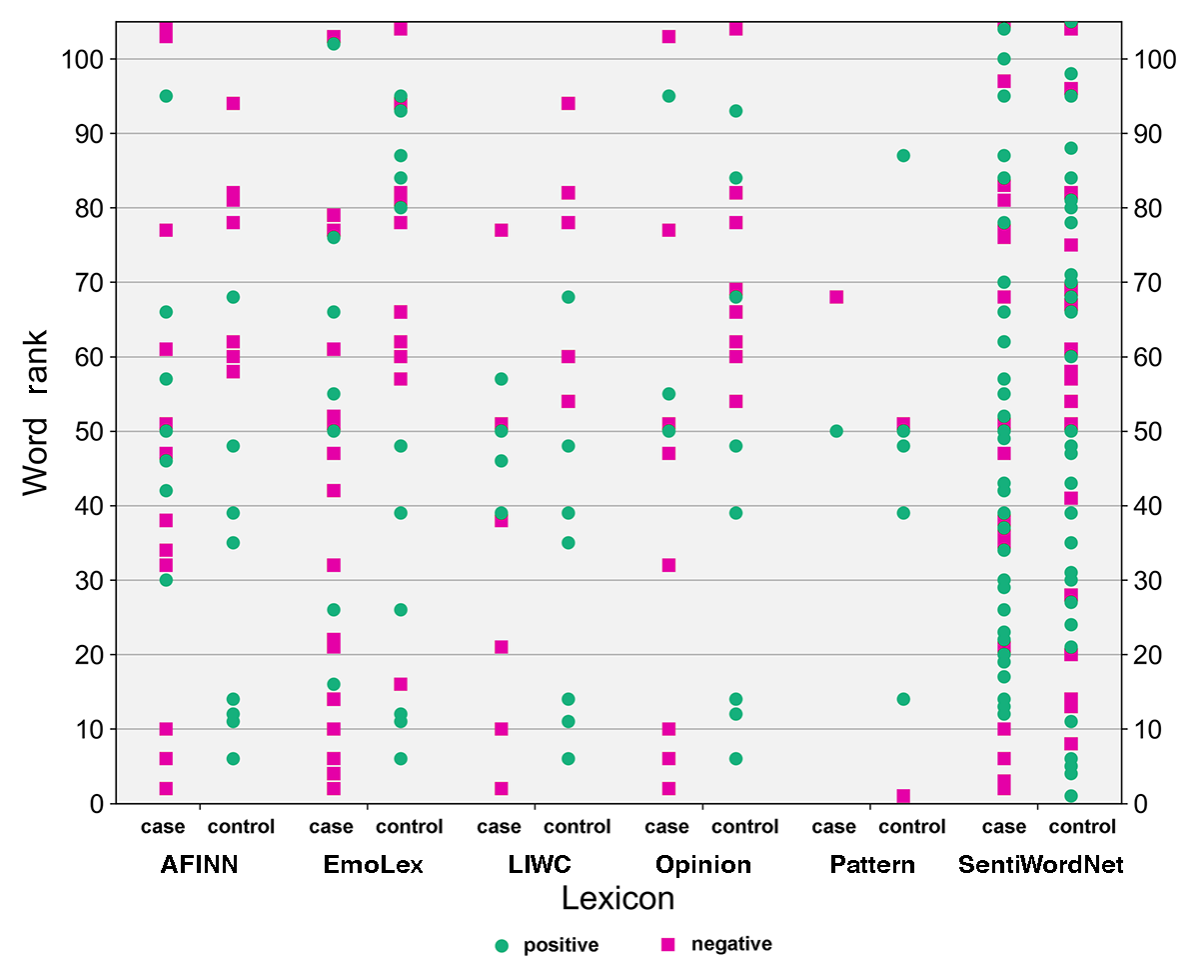
Comparative sentiment lexicon coverage of top 100 ranked words for the suicidal case and nonsuicidal control subcorpora.

Finally, we examined the distribution of sentiment among the top-ranking representative keywords for each subcorpus (sentiment coverage). Figure 1 shows the ranks of the top 100 keywords each lexicon contains for the case and control subcorpora. In addition to plotting the ranks of words featured in each lexicon, we also indicate, through color and shape coding, the polarity associated with each term.

In terms of sentiment coverage, AFINN, EmoLex, LIWC, and Opinion mark a clear distinction between the top case and control keywords. These lexicons assign negative sentiment to high-ranking case keywords (eg, *harm* [ranked third], *risk* [11th], *kill* [52nd], and *pain* [78th]) and positive sentiment to top control keywords (eg, *please* [seventh], *calm* [40th], and *pleasant* [49th]), and negative also to certain high-ranking control keywords (eg, *aggressive* [61st], *illness* [63rd], and *anxiety* [83rd]).

Only 2 high-ranking keywords for cases appeared in the Pattern lexicon: these were *safe* [51st], which was the only one of the top 100 ranked words consistently found for cases across all 7 lexicons, and *past* [68th], which only appeared in Pattern and was ascribed a negative polarity (further discussed in the *Discussion* section). *Calm* [40th] and *pleasant* [49th] were the only top 100 keywords found consistently for controls across all 6 lexicons, and these were ascribed a positive polarity by all except SentiWordNet. This unexpected assignment of sentiment (the adjective *calm* is given a heavily negative score in SentiWordNet, whereas *anxious*, *borderline*, *cutting*, and *concern* are positive) highlights the importance of studying the underlying assumptions in off-the-shelf tools and their potential implications when applying them for a new use case.

For SentiWordNet, sentiment of top keywords is mixed, with a higher proportion of positive sentiment keywords in both subcorpora, although it assigned more negative sentiment for controls and for a greater proportion of the high-ranked keywords. This shows that despite having a larger lexical coverage, the sentiment coverage of this lexicon may not be sufficiently consistent to reliably distinguish the 2 populations.

It is important to note that 51 of the top 100 keywords for the case subcorpus were not identified by any of the lexicons. These included *self, staff, said, alcohol,* and *a&e*, all in the top 10 (Table 4), as well as further highly clinically relevant (although not necessarily sentiment bearing) words such as *paracetamol* (ranked 25th, FreqDiff=524.6, FreqRatio=4.5), the abbreviation *od* (used variably in psychiatry to mean either *overdose* or *omne in die* [once a day] with respect to medication; ranked 29th, FreqDiff=498.2, FreqRatio=2.2), *ambulance* (ranked 57th, FreqDiff=340.9, FreqRatio=3.3), the plural form *overdoses* (ranked 68th, FreqDiff=314.0, FreqRatio=7.6), and the acronym *dsh* (deliberate self-harm; ranked 83rd, FreqDiff=275.1, FreqRatio=3.4). The frequency ratio of these words shows that they were many times more frequent in suicide-related case notes than in the control corpus. Over the entire list of case keywords, only 33.35% (787/2360) were assigned a sentiment value by at least one of the lexicons. Furthermore, 51 of the top 100 control keywords were also absent from all lexicons, many of which pertain to correspondence (eg, *mr*, *appointment*, and *fax*). We refer the reader to Multimedia Appendix 2 for further details.

## Discussion

### Implications for Suicide Risk Assessment Lexicon Development

The list of representative keywords extracted from our corpus shows that the notion of sentiment generally adopted in the field of NLP is not the most appropriate semantic category for identifying terms that typify case notes of suicidal patients. Many of these terms do not carry an obvious negative or positive polarity, as defined in the tested sentiment lexicons.

Our analysis also showed that there is a need for further analysis of the assignment of sentiment polarity by these tools when applied on new use cases.

Furthermore, many of the keywords we identified as representative of suicide-related case notes were *neutral* with respect to sentiment, which is expected, and representative case keywords extracted in our study indicate that they are distinct from control keywords, but not all such terms would necessarily be sentiment bearing.

Our results show that these sentiment lexicons built using validated lexical resources, such as dictionaries or thesauri (eg, EmoLex), had higher combined precision and recall results than those derived from semiautomatic processes over large open-domain text corpora (eg, Opinion, built by web crawling).

### Guidelines for Building Sentiment Lexicons for Suicide Risk Assessment

Following the work of Deng et al [24], one solution to the unsuitability of general-domain lexical resources for the clinical domain consists of defining the notion of sentiment for the analysis of clinical texts, and in the present case, of mental health (Guideline 1). This could allow the assignment of polarity to terms that do not feature in general-purpose lexical resources. In the case of suicide risk assessment, this might include the assignment of negative polarity to terms such as *a&e*, *overdose*, *alcohol*, *dsh*, and *plan*, which were not assigned a polarity value by the lexicons we tested.

In light of our results, a suggested strategy for building a suicide risk assessment lexicon may be to use corpus word frequencies as a guide to inclusion of words in a lexical resource that would remain agnostic with respect to sentiment (Guideline 2) and instead labeling terms as *trigger* or *risk factor* words (Guideline 3). Such a strategy would avoid the problem of assigning sentiment to words which, although highly representative of suicide-related texts, do not have an obvious *sentiment* value. This would also obviate the need to assign a polarity to terms that may be ambiguous in the sentiment they express, being either positive or negative depending on context (eg, *low* [emotion] vs *low* [risk]), although the more general problem of polysemy remains.

For clinically relevant terms, specialized psychiatric dictionaries or health care terminologies could be beneficial in creating a targeted lexical resource for suicide risk assessment (Guideline 4). For example, certain risk factors for suicide (eg, previous suicide attempts, depression, and substance misuse) and protective factors (eg, effective clinical care, family, and community support) are already well-known clinical features. Therefore, these concepts and associated terms should be reflected in any lexicon aiming to identify periods of increased suicide risk in clinical notes. One caveat that must be kept in mind is that many terms contained in specialized clinical terminologies are not written in EHRs by clinicians [46], meaning that term selection should be carried out by domain experts with a general awareness of typical target corpora.

Automated approaches to extracting terms from large corpora have become common in the field of NLP, including the creation of sentiment lexicons [47-49]. These techniques provide a means to increase the coverage of relevant terms, although it is preferable to implement some mechanism to ensure that the criterion of relevance is respected. Incorporating a domain-specific corpus-based notion of term *representativeness* into automatic lexicon induction procedures [50] is one way of refining term selection, filtering out terms that are deemed to be nonrepresentative (Guideline 5). Furthermore, a manual validation by domain experts (Guideline 6), where feasible, would further serve to ensure the precision of the extracted terms and could also be used to assign additional semantic categories such as sentiment.

Summary of guidelines is as follows:

1. Define the notion of sentiment for the clinical domain
2. Use corpus word frequencies as a guide to inclusion of words in a lexicon
3. Label terms as *risk factor* or *trigger* rather than sentiment-bearing
4. Use specialized dictionaries and/or health care terminologies as a source
5. Incorporate domain-specific corpus-based notion of representativeness into automatic lexicon induction techniques
6. Manual validation by domain experts

### Summary and Limitations

Examining our data using the methods of corpus linguistics revealed statistically significant differences between the keywords used in EHR notes preceding an admission for attempted suicide and those from control periods not associated with such an attempt. Themes included hospitalized suicide attempts, self-harm, and alcohol. Coverage of these keywords by the general-purpose sentiment lexicons we reviewed was varied. Although lexicon size was a determining factor in overall coverage, the largest resource, SentiWordNet, did not distinguish the 2 subcorpora as well as some of the smaller resources, namely, AFINN, EmoLex, and Opinion, once both keyword rankings and sentiment were taken into consideration. Similarly, EmoLex and AFINN had wider coverage of relevant keywords than Opinion, which is the largest of the 3 resources. This may be partly a consequence of the original sampling strategy used to select words to construct sentiment lexicons. Both EmoLex and AFINN were built on top of existing general-purpose dictionaries, whereas Opinion was created semiautomatically by crawling product reviews on the internet. As a result, the vocabulary of the latter may be more specific to that domain, whereas the 2 former lexicons are likely to be more generic in their terminology, meaning they may adapt slightly better to different domains. The same 3 lexicons also showed the most discriminating assignment of sentiment polarity between the case and control keywords. Although many of the terms contained in these resources can be said to convey appropriate sentiment values (eg, *anxiety* is negative and *pleasant* is positive), there are also certain terms for which this is less obvious, at least in the context of EHR text related to suicide risk. For example, *ward* is assigned negative sentiment by SentiWordNet, whereas *thoughts* are assigned positive sentiment. The word *plan* is assigned positive sentiment by EmoLex, whereas *call* is negative. Annotating word polarity in a noncontextual manner, especially without appropriate part-of-speech disambiguation (only 2 of the resources we tested contained entries with part-of-speech information), could lead to biased analyses in downstream modeling of new use cases. Clinical texts are intended to be written in an objective style, rather lacking what one might generally term *sentiment*, although in reality this may not always be the case. Many of the most highly relevant terms identified by our approach (eg, *a&e*, *overdoses*, and *alcohol*) do not fall into what might typically be termed a sentiment category but rather belong to categories of risk factors, whereas other identified terms are more sentiment bearing.

These observations lead us to concur with the conclusions of previous research [21-24] that domain-specific resources need to be developed for the analysis of clinical texts. We have attempted to provide insight into why this might be and what information such resources might need to include to address the task of suicide risk assessment through the analysis of clinical notes.

Our study has some limitations. First, the corpus was not constructed according to a deliberate sampling strategy but is the result of a 1:4 case-control selection ratio, which is typical in epidemiology. Completed and attempted suicide is much rarer than our sample suggests. Furthermore, the documents were not sampled according to type. This may have led to a preponderance of letters in the control corpus, as suggested by the most frequent keywords. The distribution of documents between patients also differs between the case and control subcorpora. Cases have, on average, almost 3 times the number of documents as controls, which is reflective of more frequent contact with mental health services. Consequently, the resulting corpus does not necessarily fulfill the criteria of representativeness and balance generally recommended in corpus linguistics.

We also acknowledge that our normalization of sentiment values for the sake of comparison does not necessarily reflect the actual quantity of sentiment assigned by all lexicons and invite the reader to refer to previous studies where *raw* sentiment scores are compared [20-22]. It is also worth noting that previous studies have shown that emotions, such as happiness expressed in social media posts, may vary with population demographics, geographical location [51,52], movement, and residency status in an area [53]. Although our work has focused on clinical texts instead of social media, such factors may have influenced our results; however, we have not controlled for this. This represents a caveat concerning the generalizability of our results to clinical populations in other geographical areas with potentially different sociodemographic configurations.

Finally, we only examined keywords that were common to both subcorpora. As a consequence, certain keywords typical of suicidal case notes only appearing in the case subcorpus may have been missed out, although we did find keywords appearing in only 1 subcorpus to be relatively infrequent compared with those we did examine.

### Conclusions

This work makes several contributions to the study of sentiment in suicide risk assessment.

First, our corpus of clinical notes drawn from a case-control study of suicidal and nonsuicidal hospital admissions is, to our knowledge, a novel use of EHRs in this area.

Second, by applying methods of corpus linguistics, we identified 2 lists of keywords: the first representative of the clinical notes of patients leading up to a hospitalized suicide attempt and a second for those who made no such attempt. We used these lists of keywords to gauge the coverage of 6 sentiment lexicons over our corpus, using a number of measures, including information retrieval metrics, which we adapted for the purposes of our evaluation. Our study provided a novel examination of the content of these lexicons and their implications in relation to sentiment analysis as well as deeper insights into the characteristics of terms that distinguish suicide risk cases from controls in EHR text. Furthermore, we found that these general-domain resources assign polarity values that are sometimes not clinically meaningful or consistent with clinical judgments.

Finally, based on the outcomes of our study, we have suggested a set of simple and clear guidelines to facilitate the creation of more useful lexical resources for those seeking to assess risk of suicide through the analysis of clinical notes. Such targeted lexicons have the potential to advance research into the use of EHRs for the study of suicide risk in clinical populations by providing discriminative features for use in both rule-based and machine learning classification systems.

## Appendix

Multimedia Appendix 1 Technical details of data and analyses.

Multimedia Appendix 2 Top 100 case and control keywords and associated polarities per lexicon.

## Abbreviations

CRIS: Clinical Record Interactive Search
EHR: electronic health record
Freq: frequency
FreqDiff: frequency difference
FreqRatio: frequency ratio
ICD: International Classification of Diseases
LIWC: Linguistic Inquiry and Word Count
NLP: natural language processing
