# Supplementary material for: Using General-purpose Sentiment Lexicons for Suicide Risk Assessment in Electronic Health Records: Corpus-Based Analysis
Source: JMIR Med Inform. 2021 Apr 13;9(4):e22397. doi: 10.2196/22397 (PMC8080148; doi:10.2196/22397)
Supplement: Multimedia Appendix 1 [file medinform_v9i4e22397_app1.docx]

# Technical Appendix

## EHR corpus – distribution of documents per patient

The distribution of documents across patients is non-normal for both case and control sub-corpora. Table 1 provides descriptive statistics for the number of documents per patient and Figures 1 and 2 plot the distributions. Cases have, on average, almost three times as many documents as controls, reflecting more regular contact with mental health services. The non-normal distribution of documents motivated the use of the non-parametric Mann Whitney U test to determine the statistical significance of word frequency differences between the two sub-corpora.

Table 1. Distribution of documents per patient

|  | **case** | **control** |
| --- | --- | --- |
| count (patients) | 2,913 | 14,727 |
| mean (doc/patient) | 24.51 | 8.55 |
| std | 66.44 | 20.90 |
| min | 1 | 1 |
| 25% | 2 | 2 |
| 50% | 6 | 3 |
| 75% | 16 | 6 |
| max | 1,509 | 344 |

Figure 1: Distribution of documents per patient for suicidal cases


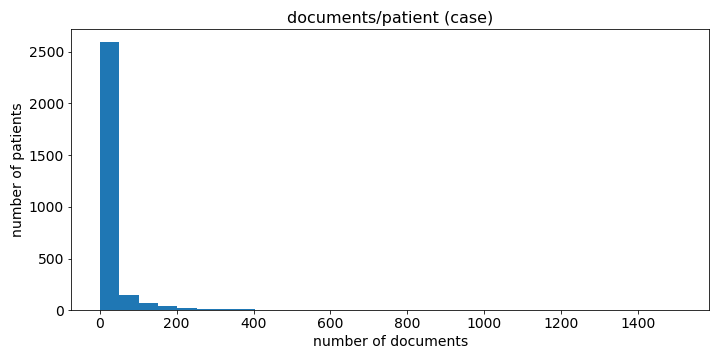


Figure 2: Distribution of documents per patient for non-suicidal controls


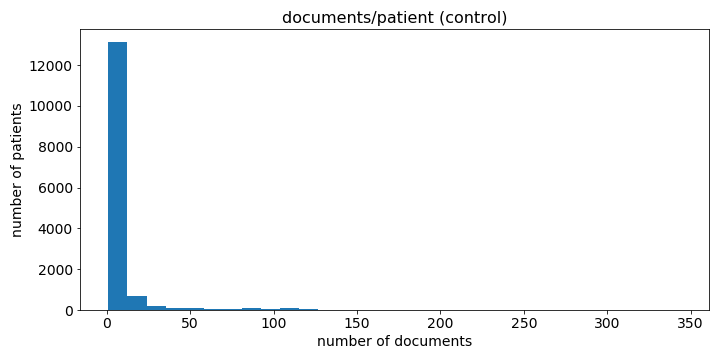


The distribution of word tokens between patients is more indicative of the “amount” of text actually present in the sub-corpora of each population.

Table 2. Distribution of word tokens per patient

|  | **case** | **control** |
| --- | --- | --- |
| count (patients) | 2,913 | 14,727 |
| mean (doc/patient) | 5,517.42 | 1,707.35 |
| std | 13,937.45 | 4,225.59 |
| min | 1 | 1 |
| 25% | 522 | 194 |
| 50% | 1,443 | 501 |
| 75% | 4,570 | 1,395 |
| max | 290,945 | 141,083 |

As for documents, the distribution is non-normal (see Figures 3 and 4). Cases have, on average, three times the number of tokens than controls.

Figure 3: Distribution of word tokens per patient for suicidal cases.


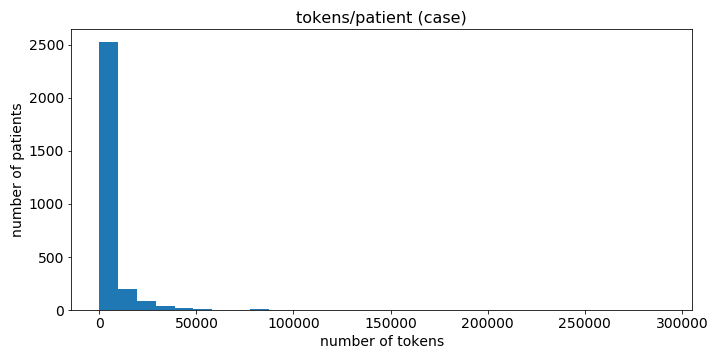


Figure 4: Distribution of word tokens per patient for non-suicidal controls.


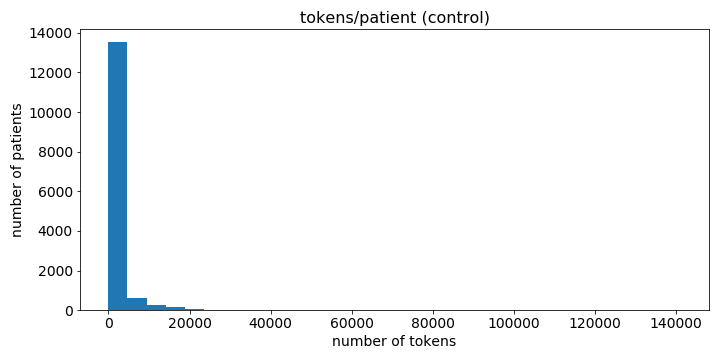


## Sentiment lexicons

Below are details of the characteristics of each of the lexicons used in the study, including modifications made to facilitate comparison.

- **AFN:** AFINN [36], is a manually labelled lexicon of polarity-conveying word forms. This lexicon was initially constituted from a list of obscene words gleaned from two research works in psychology and media studies. This list was then manually expanded with terms from Twitter, as well as other online resources, such as Internet slang dictionaries and affective word lists. Each entry in the lexicon is assigned an integer polarity score between -5 (most negative) and 5 (most positive).
- **EMO:** The NRC Emotion Lexicon [37] (commonly known as EmoLex), contains inflected word forms associated with 8 basic emotion categories, as well as negative and positive polarities. Target words were sourced from the Macquarie Thesaurus [41], a subset of the WordNet-Affect lexicon [42] and the General Inquirer [43]. Terms extracted from the thesaurus were selected according to their frequency in the Google n-gram corpus [44] to ensure coverage of most frequent terms. Polarity and emotion values were then assigned manually via crowdsourcing. Each entry in the lexicon has a binary value (0 or 1) for each of the 10 categories. An entry may have multiple values (e.g. anger, sadness and negative). We removed all entries in the lexicon where all category values were 0 (neutral), retaining only those with values of 1 for “positive” or “negative”.
- **LWC:** Linguistic Inquiry and Word Count (LIWC) is a lexicometric software tool that counts words in “psychologically meaningful categories” [38, p1]. It was originally developed to analyse personal narratives to derive insights on the writer’s psychology and the correlation between word use and health outcomes. LIWC is packaged with a lexicon in which terms are represented as either inflected word forms or regular expression-like patterns (e.g. the pattern ‘hesita*’ matches ‘hesitation’, ‘hesitating’, ‘hesitant’, ‘hesitated’, etc.). The lexicon includes categories for positive emotion and negative emotion, which are the categories of words we retained for this study. The terms in the subjective categories of the LIWC lexicon were manually sourced from unspecified “dictionaries, thesauruses, questionnaires, and lists made by research assistants” [38, p27] and categorised by human judges. We used the 2015 version of the lexicon.
- **OPN:** The Opinion lexicon [9] contains inflected word forms sourced by web-crawling product reviews. Polarity values for each entry were assigned automatically via comparison with WordNet [35]. Each entry in the lexicon is labelled as either positive or negative.
- **PAT:** Pattern [39] is a stand-alone NLP package with a sentiment analysis component. It uses a lexicon based on a subset of WordNet, containing word lemmas along with their part-of-speech, annotated with a sentiment score between -1 and 1. Pattern applies contextual processing of negation (e.g. not happy) and adverbial modification (e.g. very happy) and provides a single polarity score for each input text. We applied the Pattern lexicon without contextual processing, removing all entries with a polarity score of 0 (neutral).
- **SWN:** SentiWordNet [40] is a lexicon that is also based on WordNet. Each lemma (with its part-of-speech) is associated with one or more sets of synonyms (synsets) and each synset, in turn, has a positive and a negative score (both between 0 and 1). These polarity scores were assigned automatically through supervised machine learning classification. For each entry in the lexicon, we retained the higher of the two scores as the term’s sentiment and chose a random sentiment in case positive and negative scores were equal. SentiWordNet contains both single- and multi-word expressions (MWEs). For simplicity and comparison with the other lexicons, which do not contain MWEs, we only took into account single-word entries. We looked up terms using the NLTK interface. For entries with multiple synsets, we chose the first one (the “most common” sense according to the lexicon’s accompanying documentation).

## Corpus analysis

A variety of methods have been used to calculate and compare word frequencies across corpora (e.g. log likelihood and chi-squared tests). However, many of these treat a corpus as a “bag-of-words”, which does not account for the uneven dispersion of words that is inherent to collections of texts (see [34] for further discussion).

Our text corpus contains two different sub-corpora that we wished to characterise by finding their most representative keywords. Each of these sub-corpora contains documents for different patients and on different days. For cases, documentation is during the period prior to an admission for attempted suicide, while for controls there is no such admission. A word that appears very frequently in only a section of the corpus (e.g. for only a single patient, or on a given day) cannot be deemed “representative” of the corpus as a whole, unlike words that are more evenly distributed across the documents in the corpus.

Following recommendations from previous research in corpus linguistics [31,32,33], and given the non-normal distribution of documents between patients, we adopted the non-parametric Mann Whitney U test (MWU, also known as the Wilcoxon rank-sum test) to calculate the significance of word frequency differences across our two sub-corpora as this test accounts for potential variation in the dispersion of words across documents.

To create a list of keywords for each sub-corpus, we applied the following steps:

1. Calculate word frequencies for each sub-corpus, as words-per-million (wpm).
2. Calculate the difference in frequencies for each word between sub-corpora.
   For a given word w:

| $FreqDiff\left( w \right)=Freq_{case}\left( w \right)-Freq_{control}\left( w \right)$ |
| --- |

Thus, words with FreqDiff(w) > 0 are case keywords and those with FreqDiff(w) < 0 are control keywords.

1. Calculate word frequency ratios to determine the relative prevalence of words between sub-corpora. The ratio is simply the maximum frequency of a word in the two sub-corpora divided by the minimum of the two values.

$$FreqRatio\left( w \right)= \frac{max(Freq_{case}\left( w \right), Freq_{control}\left( w \right))}{min(Freq_{case}\left( w \right), Freq_{control}\left( w \right))}$$

2. Test significance of word frequency differences with MWU. Remove words with *P* value above a certain threshold (we use *P*>.000001, the most conservative value used in [36] to only retain the most significant words).
3. Sort list by FreqDiff to rank keywords.

## Lexicon analysis

**Global coverage:** For lexicons containing lemmas, matching was done on the lemmatised corpus (e.g. ‘overdose’ matched ‘overdose’, ‘overdoses’, ‘overdosed’ and ‘overdosing’). For lexicons of word forms, this was calculated on the tokenised word forms. This provided a general comparison of lexical coverage across all words.

**Keyword coverage:** To determine lexicon keyword coverage, we calculated weighted precision, recall and F-score for each lexicon using the following equations:

$Pw= \frac{TPw}{TPw+FPw} Rw= \frac{TPw}{TPw+FNw} Fw=\frac{2\left( P+R \right)}{\left( P*R \right)}$

We define TP_w_ as the summed absolute frequency difference (FreqDiff) of all case keywords present in the lexicon, FP_w_ is the summed absolute frequency difference of all control keywords in the lexicon and FN_w_ is the summed absolute frequency difference of all case keywords not present in the lexicon. Introducing the (ranked) differences in word frequencies as a weighting, as opposed to the similar unweighted metrics, rewards inclusion of high-ranking case keywords and the exclusion of control keywords while penalising the inclusion of control keywords and the exclusion of case keywords.

## Sentiment lexicon coverage

For the control sub-corpus, we calculate a raw percentage, as well as an adjusted percentage that compensates for the difference in word type ratio (1:~1.5) and word token ratio (1:~1.6) between the two sub-corpora.
